# Effect of Affective Personality Information on Face Processing: Evidence from ERPs

**DOI:** 10.3389/fpsyg.2016.00810

**Published:** 2016-05-31

**Authors:** Qiu L. Luo, Han L. Wang, Milena Dzhelyova, Ping Huang, Lei Mo

**Affiliations:** ^1^Center for the Study of Applied Psychology and School of Psychology, South China Normal UniversityGuangzhou, China; ^2^Guangdong Provincial Key Laboratory of Mental Health and Cognitive Science, South China Normal UniversityGuangzhou, China; ^3^Psychological Sciences Research Institute and Institute of Neuroscience, Universite Catholique de LouvainLouvain-la-Neuve, Belgium

**Keywords:** person perception, face processing, personality, ERPs

## Abstract

This study explored the extent to which there are the neural correlates of the affective personality influence on face processing using event-related potentials (ERPs). In the learning phase, participants viewed a target individual’s face (expression neutral or faint smile) paired with either negative, neutral or positive sentences describing previous typical behavior of the target. In the following EEG testing phase, participants completed gender judgments of the learned faces. Statistical analyses were conducted on measures of neural activity during the gender judgment task. Repeated measures ANOVA of ERP data showed that faces described as having a negative personality elicited larger N170 than did those with a neutral or positive description. The early posterior negativity (EPN) showed the same result pattern, with larger amplitudes for faces paired with negative personality than for others. The size of the late positive potential was larger for faces paired with positive personality than for those with neutral and negative personality. The current study indicates that affective personality information is associated with an automatic, top–down modulation on face processing.

## Introduction

Personality is proposed as one of the principal affective knowledge during social interaction, determining our evaluations and actions (Willis and Todorov, 2006; Lorenzo et al., 2010). It has been well-documented that information of personality can be acquired via facial appearance in a spectacularly short period of time (Bar et al., 2006; Todorov et al., 2008). Specifically, trustworthiness from face can be determined at first glimpse within about 100 ms. Moreover, personality knowledge can also be inferred quickly from verbal descriptions (Willis and Todorov, 2006). The experiment was modeled upon behavioral studies showing that even a single behavior description is sufficient to trigger trait inference (Todorov et al., 2007; Collins and Olson, 2014; Schneid et al., 2015). This trait inference occurs spontaneously even in the condition of constrained cognitive resources (Todorov and Uleman, 2003). The current study was interested in whether the high-level affective personality information could influent the perception of that individual, especially the perception of faces.

Many evident suggest that affective personality information impact facial recognition. It was found that judgments of physical attractiveness were affected by personality and character information, with more positively described stimulus persons being rated as more physically attractive (Gross and Crofton, 1977). In another behavioral study, photo reproductions were present with descriptive sentences providing social character information about the depicted individuals. It was found that faces of cheaters were preferentially recognized by participants (Mealey et al., 1996; Oda, 1997). Differently, Baker et al. (2013) present moral information in vignettes in which described the target’s behavior. Face-vignette pairings were manipulated to determine the different personality impressions for disparate faces. Then participants were asked to identify the target faces. Faces were found to recall as having less trustworthy features following exposure to information about immoral behavior. The facial identification results suggested that behavioral information varying in morality influenced facial feature memory (Baker et al., 2013). The enhanced recognition for faces giving an impression of untrustworthiness was suggested to reflect an interaction between the avoidance-related insular region and the encoding-related hippocampal region (Tsukiura et al., 2012).

Furthermore, several experiments demonstrate that social affective information modulates facial processing. Gossip as a form of affective information was found to affect vision of face, with faces paired with negative gossip having longer visual consciousness. Crucially, the modulation of the affective knowledge on face was found occurring in the early sensory processing period. In an event related potentials (ERP) study, biographic information was assigned to famous or initially unfamiliar faces. The affective personal knowledge was found to modulate the electro-cortical response at posterior sites between 200 and 300 ms only for well-known faces, lasting to 500 and 600 ms at parietal sites for well-known and initially unfamiliar faces (Abdel Rahman, 2011; Wieser et al., 2014). Similar procedure was applied in the study of Suess et al. (2015), it was found that negative social knowledge could bias the perception of both well-known and newly learn faces indexing respectively in the time windows 200–350 and 300–350 ms. Although these investigations demonstrate the temporal dynamics of affective knowledge effects on face processing, they leave some controversies for future research. Firstly, complex personal information (e.g., name, age, vocation, interests, or personality) was integrated in a single biography in the previous research, therefore making it difficult to segregate the influence of personality information on face processing. Moreover, the isolation between learning and testing (1 or 2 days apart) in the mentioned studies led to dominant recognition for faces in the negative relative to other conditions. Then, the segregated early neural response among faces paired with different affective biographhical in these research could not eliminate the influence of stimuli familiarity (Tanaka et al., 2006; Dichter and Belger, 2007). Whether affective personality could modulate face processing. Whether, the effect of personality information could directly index by early ERP components of face processing. Given these issues, we applied simple sentence to present social information, and used face-sentence paired learning to determine the influence of positive, neutral, or negative personality information on observers’ neural representation of target faces in the present study. Also, ERP testing was implemented just after learning to maintain the stimuli association.

To identify the neural correlates of affective personality influence on face processing, ERPs with high temporal resolution were preferentially applied to investigate the neural activities implicated in this process. Base on previous evidence, early posterior negativity (EPN) and late positive potential (LPP) are two primary components involved in face processing and affected by socially affective information. EPN is an enhanced negativity at temporo-occipital sites between 200 and 350 ms post-stimulus (Werheid et al., 2007). It was suggested to reflect a second stage of cognitive decoding of emotional stimuli, which is associated with more strategic processing (Schupp et al., 2004). So far, EPN was deem as the earliest component representing personal information influence on facial perception (Wieser et al., 2014). Additionally, (parietal LPP) which starts approximately at 400 ms after stimulus onset was supposed to reflect a high-level, cognitive elaboration of motivationally significant stimuli after the stimulus categorization was completed (Ritter and Ruchkin, 1992; Schupp et al., 2006). Moreover, this component has been associated with emotional processing of facial stimuli (Hajcak et al., 2009; Suess et al., 2015) with an increased LPP amplitude for emotional compared to neutral stimuli (Schupp et al., 2000; Kissler et al., 2009). It was found that LPP was larger for faces associated with negative information than others (Abdel Rahman, 2011). In the present study, analyses also include two early components related to facial perception in order to examine the early effect of affective personality. One is early positivity, P1 peaking at about 100 ms post-stimulus over temporal–occipital sites, This component has been suggested to index automatic processing of facial characteristics (Rossion and Caharel, 2011). Yet, this early component could be influenced by low-level characteristics, typically not controlled in such studies. Another component, more reliably associated with face perception is the N170. This negative deflection, peaking at occipito-temporal electrode sites at approximately 170 ms post-stimulus onset, is triggered by faces rather than by cars, hands, furniture, or scrambled faces (Bentin et al., 1996). N170 was argued to reflect the initial activation of face representations associated with the categorization of the stimulus as a face (Eimer, 2000).

To this end, we investigated the influence of affective personality information on face processing indexed by N170, EPN and LPP. More specifically, the neural responses to faces paired with positive, neutral, and negative personality information were assessed via ERPs, using a study-test paradigm. According to previous studies, it was expected that the early ERPs evidenced by N170, EPN and the late component LPP would differ for faces described as having diverse personality information, indicating that facial processing could be modulated by affective social information. In particular, faces paired with negative knowledge would elicit larger response than other faces in N170, EPN, and LPP.

## Materials and Methods

### Participants

Twenty-five right-handed volunteers (15 females) participated in this study. After receiving a complete description of the study all volunteers gave written informed consent. Participants had no prior history of neurological or psychiatric problems and had normal or corrected-to-normal vision. They were paid a fixed amount (¥30) for their participation. The study protocol was approved by the Academic Committee of the Department of Psychology at South China Normal University, which monitors the ethics of research involving human subjects. Data from two male participants were omitted from analyses because of excessive artifacts, with more than one-third of the trials being rejected for two or three conditions. This left data from 23 participants with an average age of 21.78 years (*SD* = 1.40) for further analysis. For these 23 participants, the average number of remaining trials for the positive personality condition was 36.90 ± 3.61; for the neutral personality condition, 35.35 ± 3.61; and for the negative personality condition, 33.74 ± 3.44.

### Methods

#### Facial Images

**FIGURE 1:**
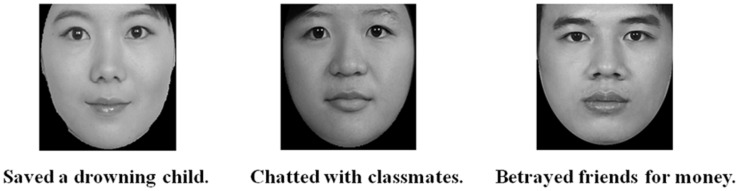
**Examples of experiment stimuli**.

One hundred photos of Chinese faces were obtained from the internet and from the native Chinese Facial Affective Picture System (CFAPS; Lu et al., 2005). These images were unfamiliar to the participants, and included no film stars, well-known musicians, or other celebrities. All faces were frontal view and forward eye-gaze. The photos were cropped to remove hair and ears, leaving only a facial mask. All stimuli were converted into grayscale images with dimensions of 202 × 225 pixels on a black background (please see **Figure 1**). 12 male faces and 12 female faces that had average attractiveness (base on ratings results of 78 participants) were selected for the ERP experiment, with no significant score difference between male and female faces. Moreover, the arousal ratings for selected 24 faces were collected from independent 29 participants, with no score significant difference between male and female faces. The expressive ratings from another 15 participants indicated neutral or near-neutral expression for these 24 faces.

#### Descriptions

We created 100 short sentences describing hypothetical past actions that varied from very negative to very positive. These sentences were 5–7 words in length and used only familiar words. A pilot study with 37 participants confirmed that the ratings for the actions ranged from very negative to very positive and were widely distributed across a nine-point scale. Base on the rating results, eight sentences that had high ratings, eight sentences that had average ratings and eight sentences that had low ratings were selected for use in the ERP experiment (Supplementary Material). The average goodness ratings among these three kinds of sentences differed significantly from each other, *ps* < 0.001.

Given the relation between stimuli arousal and LPP response (Hajcak et al., 2006), arousal ratings for selected 24 describing sentences were collected using the nine-point Likert scale from independent 29 participants (20 females; 21.21 ± 1.90 years). The positive sentences were reliably higher on arousal ratings than negative (*p* = 0.002) and neutral (*p* < 0.001) sentences. The arousal ratings for negative sentences were also significantly larger than neutral sentences, *p* < 0.001.

### Procedure and Design

All participants were seated comfortably in a dimly illuminated, acoustically and electrically shielded room. Stimuli were presented at the center of a monitor placed at eye level 100 cm in front of the participant. The experiment included two sessions in order to reduce the participants’ memory load and to make sure that all provided materials could be remembered by the participants. Each session presented 12 faces paired with 12 sentences.

Participants were instructed to join a memory experiment that comprised a learning phase, two testing phases and a gender judgment task phase for each of the two experiment sessions. In the learning phase, participants viewed a target individual’s face along with a sentence describing either the positive, neutral or negative behavior below the face stimulus (please see **Figure 1**). These faces and sentences were paired randomly across participants, and were repeated four times during the learning phase. Moreover, the face-sentence pairs were matched for gender of the faces and the types of descriptive sentences. That is, each type of sentence was paired with an equal number of male and female faces. The presentation time of stimuli, with a maximum of 2000 ms, was determined by the response of participants. The subsequent memory test (the first memory test) was administrated to ensure that association between faces and personality information had been established for each participant after the learning phase.

In each of the 12 testing trials, participants had to indicate which of the two sentences on the screen described the right behavior related to the face presented at the center of the screen. All these sentences and faces were the same as those in the learning task. Only participants who passed the memory test with higher than 90% accuracy would continue to next phase, and the others would repeat the learning phase. A post-experiment memory test (the second memory test) was also conducted to examine the memory effect after a delay. Between these two memory tests, participants were asked to perform a gender judgment task in which they classified the target faces as male or female as quickly as possible by pressing one of two keys. Correct responses on this task were not based on personality information; therefore, any processing discrepancies among different kinds of faces would reflect an automatic influence of affective personality information. Face stimuli were presented in a fully balanced pseudorandom order to ensure that proportionate numbers of each type of trial appeared over the 60 trials of every session. Each target face was presented five times in this task. Therefore, there were in total 120 trials for the whole experiment and 40 trials for each condition.

**FIGURE 2:**
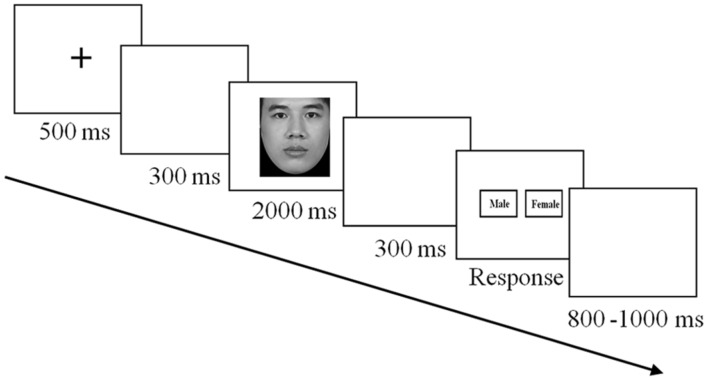
**Sequence of events in the gender judgment task**.

For the gender judgment task, each trial started with the presentation of a fixation cross for 500 ms, followed by a blank screen for 300 ms. The target face was presented for 2000 ms. In this period, participants were asked to focus on face viewing. Then a blank screen was displayed for 500 ms, after which a response interface was presented. Participants indicated the gender of the target face by pressing the ‘F’ or ‘J’ key with either their left or right index finger; the assignment of options for indicating the correct answer was random across trials. This was followed by a blank screen, which lasted between 800 and 1000 ms (please see **Figure 2**). Statistical analyses were conducted on measures of electroencephalographic activity during the face presentation.

### EEG Recording and Data Analysis

An elastic cap containing 64 electrodes was used to record EEG activity during the whole experiment (Neuroscan, Inc., Charlotte, NC, USA), with the reference placed on the end of nose (Churches et al., 2010, 2014). Bipolar horizontal and vertical electrooculograms (EOGs) were recorded simultaneously to monitor eye movements and blinks. The sampling rate was 1000 Hz. The inter-electrode impedance was maintained below 5 kΩ. The EEG and EOG signals were amplified and digitized with a band-pass of 0.01- to 100-Hz. Off-line analysis was performed using BrainVision Analyzer software. Ocular artifacts were corrected with an eye-movement correction algorithm, which employs a regression analysis in combination with artifact averaging (Semlitsch et al., 1986). The ERP waveforms were time-locked at the onset of facial stimuli appearance on the screen. The data were segmented into 1200 ms epochs (200 ms before stimulus onset was used as a baseline) and baseline corrected by subtracting the average activity of that channel during the baseline period from each sample. Then, experiment trials in which EEG voltages exceeded a threshold of ±80 mV during the recording epoch were excluded from the analysis. Subsequently, EEG data were filtered off-line using a 30-Hz low-pass.

**FIGURE 3:**
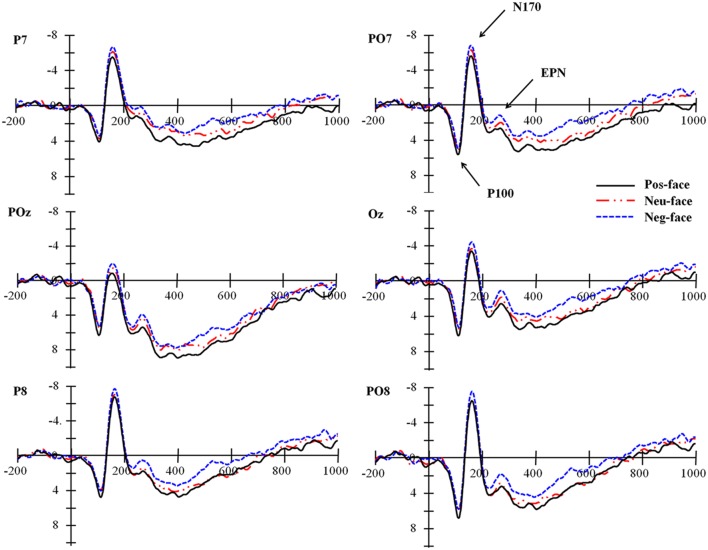
**Grand Averages at posterior sites for faces of varying personalities**. Pos, positive personality; Neu, neutral personality; Neg, negative personality.

Visual inspection of the grand averaged ERPs revealed distinct N170 and EPN, beginning nearly 130 ms post-stimulus onset at occipito-temporal electrode sites (see **Figure 3**). A recognizable P100 was also observed at those sites. According to literature about topographical distributions of facial processing, early ERP responses were mainly presented in left and right temporal regions (O’Doherty et al., 2003; Abdel Rahman, 2011). To examine this laterality effect associated with personality effect on face processing, the following six electrode sites for three ROIs were chosen for statistical analysis in the time windows of 100–140 ms (P100), 135–185 ms (N170) and 220–320 ms (EPN): P7, PO7 (ROI: left posterior); POz, Oz (ROI: midline posterior); and P8, PO8 (ROI: right posterior). LPP response was measured at different locations: C3, CP3 (ROI: left parietal); Cz, CPz (ROI: midline parietal); and C4, CP4 (ROI: right parietal) within its typical time window (400–700 ms). The average amplitudes of P100, N170, EPN, and LPP were calculated during their respective time windows and pooled across two electrodes for each ROI. Two-way repeated-measures analysis of variance (ANOVA) were performed using personality type (positive, neutral, negative), and laterality (left sites, middle sites, right sites) as independent variables and amplitudes at P100, N170, EPN, and LPP as dependent variables. In all analyses, the Greenhouse–Geisser correction for non-sphericity was applied if Mauchly’s test of sphericity was significant.

## Results

### Behavioral Results

The accuracy of the first memory test was 98.55 ± 2.03% and that of the second memory test was 97.65 ± 4.67%. A paired *t*-test indicated that these two accuracy rates were not significantly different from each other, *t*(22) = 1.00, *p* = 0.328. Then the accuracy of each experimental condition was calculated for these two memory tests respectively. No significant accuracy difference was found among three conditions either in the first or second memory test, *ps* > 0.100. Behavioral data of the gender judgment task were also analyzed. The accuracy of gender judgment was 95.69 ± 3.37%, suggesting that participants paid attention to the face stimuli during ERP recording. However, no statistical accuracy difference was found for the gender judgment of faces paired with positive, neutral, and negative personality information, *p* > 0.5. The RTs for gender judgment in different personality conditions were also not significantly different from each other, *p* = 0.991.

### ERP Results

#### P100

**Figure 3** presents stimulus-locked average ERPs for faces paired with varying personality information. The average amplitude of the P100 over the three conditions did not show a significant difference, *F*(2,44) = 2.20, *p* = 0.123. A main effect of electrode laterality was also found, *F*(2,44) = 5.10, *p* = 0.010, $\eta_{p}^{2}$ = 0.188. A pair-wise comparison revealed that P100 was smaller at left posterior than middle (*p* = 0.003) and right (*p* = 0.036) posterior sites. The interaction was not significant, *p* = 0.827. The amplitudes for different conditions are listed in **Table 1**.

**Table 1:** Mean and standard deviation of ERP amplitudes (μV).

	P100	N170	EPN	LPP
**Conditions**				
Positive personality	3.29 (2.83)	-3.40 (4.05)	3.60 (5.02)	8.00 (3.69)
Neutral personality	2.84 (2.70)	-3.79 (3.87)	3.11 (5.00)	6.97 (3.72)
Negative personality	2.53 (3.08)	-4.45 (4.65)	2.38 (5.19)	6.68 (4.44)
**ROIs**				
Left posterior	2.13 (2.38)	-4.81 (3.51)	2.06 (5.05)	6.58 (3.57)
Middle posterior	3.46 (3.09)	-1.66 (3.92)	4.29 (4.79)	7.86 (4.39)
Right posterior	3.07 (2.98)	-5.16 (4.28)	2.74 (5.18)	7.22 (3.91)

#### N170

The two-factor repeated-measures ANOVA conducted for the mean amplitude of N170 yielded a significant main effect of personality type, *F*(2,44) = 4.20, *p* = 0.025, $\eta_{p}^{2}$ = 0.160. The N170 amplitude decreased gradually from the negative to neutral and then positive personality condition (see **Table 1**). *Post hoc* tests indicated that N170 was significantly larger for the faces paired with negative personality than those with neutral (*p* = 0.044) and positive personality (*p* = 0.012). No significant difference was found between faces paired with positive personality and neutral personality, *p* = 0.340. A main effect of laterality was also found, *F*(2,44) = 21.11, *p* = < 0.001, $\eta_{p}^{2}$ = 0.490. A pair-wise comparison confirmed that N170 was more negative at left and right posterior sites than at middle posterior sites, *p* < 0.001, while the amplitude difference between left and right posterior sites did not reach statistical significance, *p* > 0.500. No significant interaction was found, *p* > 0.500.

#### Early Posterior Negativity

The EPN data mirrored the N170 data. Analysis of EPN amplitudes showed main effects of personality type, *F*(2,44) = 4.98, *p* = 0.015, $\eta_{p}^{2}$ = 0.184, and laterality, *F*(2,44) = 11.84, *p* = <0.001, $\eta_{p}^{2}$ = 0.350. The amplitude of EPN decreased gradually from negative to neutral and then to positive personality (see **Table 1**). *Post hoc* tests showed that the amplitude of EPN elicited by faces paired with negative personality was significantly larger than those with neutral (*p* = 0.029) and positive personality (*p* = 0.009), but the amplitude difference between the latter two conditions was not significant, *p* > 0.20. A pair-wise comparison confirmed that EPN at middle posterior sites was significantly smaller than that at left (*p* = < 0.001) and right posterior (*p* = < 0.001) sites, while the amplitudes for left and right posterior sites did not differ from each other, *p* > 0.20.

#### Late Positive Potential

**FIGURE 4:**
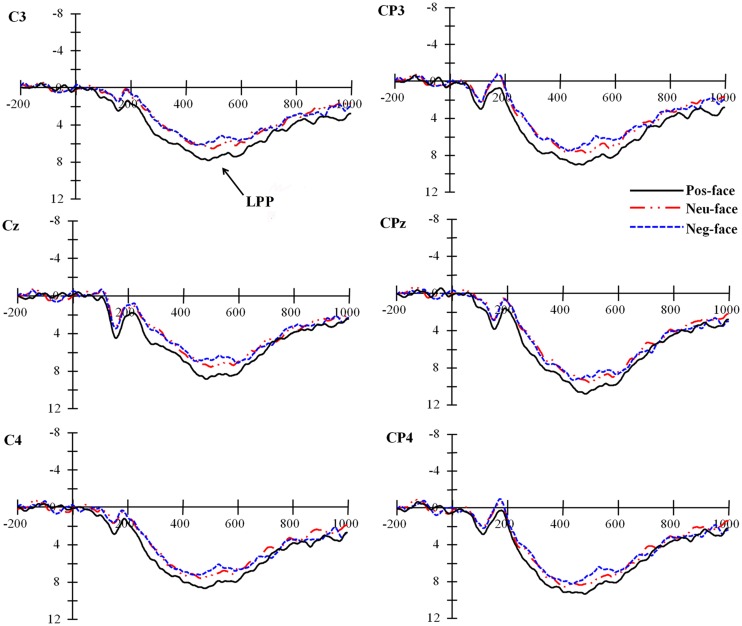
**Grand averages at parietal sites for faces of varying personalities**.

Analysis of this component showed significant main effects of personality type, *F*(2,44) = 4.51, *p* = 0.016, $\eta_{p}^{2}$ = 0.170, and laterality, *F*(2,44) = 14.14, *p* < 0.001, $\eta_{p}^{2}$ = 0.391. *Post hoc* tests indicated that the amplitudes related with faces paired with positive personality were significantly larger than those with neutral (*p* = 0.015) and negative (*p* = 0.016) personality, whereas the amplitude difference between the latter two conditions was not significant, *p* > 0.50, see **Figure 4**. The LPP amplitude was largest at middle parietal sites. *Post hoc* tests indicated that LPP at middle parietal sites was significantly larger than that at left (*p* = < 0.001) and right (*p* = 0.008) parietal sites. Moreover, the amplitude difference between left and right parietal sites also reached statistical significance, *p* = 0.018.

## Discussion

In the present study, face-sentence pairings were manipulated to identify the influence of affective personality information on face processing. The high and comparable accuracy for faces paired with either the positive, neutral or negative personality information indicated successful learning of stimuli pairs for each condition. Moreover, as expected, a short exposure to personality information influenced the neural responses to the target faces at early and late latencies. Distinctive activations specific to the affective personality information emerged as early as around 170 ms and lasted to the following periods. The results of N170 and EPN showed a similar pattern with larger amplitudes for faces paired with negative personality than those paired with neutral and positive personality. The size of LPP was found larger for faces paired with positive personality than those paired with negative and neutral personality. These findings enabled us to identify the neural mechanism of affective personality modulation on face processing.

### N170 and Initial Perceptual Encoding of Affective Stimuli

The first hint of a differential processing of faces paired with a disclosure of positive, neutral, and negative personality occurred around 170 ms. Intriguingly, our findings indicated a much earlier modulation of affective personal information on sensory processing than previous research (Abdel Rahman, 2011; Wieser et al., 2014). In previous studies, EPN was the earliest component found modulated by affective information. Yet, in such studies complex personal information was provided which potentially could have lead to the necessity of more elaborate processing, requiring longer time. The early effect of personality information on the amplitude of N170 in our experiment should not be ascribed to structural facial features or configuration, as facial attractiveness of target faces was equalized and all faces displayed a neutral expression. This claim is also supported by the absence of any difference between the faces paired with disparate personalities on P100. The visual P100 is influenced by low-level properties of visual stimuli (Rossion and Jacques, 2008). Moreover, in the present study the faces were randomly paired with behavior sentences across participants in order to eliminate the bias of stimuli combination. As the task is a simple perceptual task that neither demands person evaluation nor retrieval of personality information, it is unlikely that the N170 results are attributable to directed attention. In other words, the N170 difference among faces coupled with varying personalities should be due to a spontaneous influence from personality information, indicating specific neural correlates of a learned social information effect on early face processing. As N170 was found to correlate with activation of the fusiform face area (FFA) and the superior temporal sulcus (STS; Rousselet et al., 2004; Bentin et al., 2006), indexing face-specific structural encoding (Bentin and Deouell, 2000; Itier and Taylor, 2004), we suggest that the early neural segregation indicates an automatic, rapid modulation of facial structural perception by affective personality information.

Our findings pose some questions to the classical perspective of N170 as a solely reliable index of early encoding of facial features and configurations (e.g., Eimer and Holmes, 2007; Jacques and Rossion, 2007; Kuefner et al., 2010). It has been demonstrated in the past that N170 can be modulated by social affective information (Pizzagalli et al., 2002; Eger et al., 2003). For example, compared to neutral and positive expressions, the expression of fear or threat is related to larger N170 response (Batty and Taylor, 2003; Schupp et al., 2004). In the current research, transitory personality information modulated the size of N170, suggesting affective perspectives on N170 deflection. The current findings provide further insights regarding N170 as a component that is sensitive not only to facial features but also to non-facial information. More intriguingly, faces described as having a negative personality are given precedence in the neural processing systems during the early period, with larger N170 for the negative personality condition than for the neutral or positive personality condition. It was suspected that personality information, as a potent form of social affective learning, could produce adaptively top–down signaling to early visual mechanisms to strategically mediate the initial structural encoding of facial stimuli.

### EPN and Elaborate Perceptual Encoding of Affective Stimuli

The personality influence on facial perception was not only shown in the N170 potential but also in the subsequent EPN. The results of EPN replicate previous findings concerning affective social influence on face processing. eEPN component has been suggested to signal “emotional significance” (Schacht and Sommer, 2009). Enhanced EPN response has been observed while participants were viewing pictures of erotica, mutilation and threat (Schupp et al., 2004; Leppänen et al., 2007). EPN has also been shown to be sensitive to social characteristics (Marzi et al., 2012). For example, attributions of trustworthiness modulated the size of EPN during explicit facial trustworthiness judgments (Dzhelyova et al., 2012). Moreover, compared to neutral faces, threatening faces have been shown to be related with more enhanced EPN amplitudes (Schupp et al., 2004). Therefore, EPN was suggested to reflect enhanced perceptual encoding resulting from reflex-like visual attention to intrinsically salient stimuli (Kissler et al., 2007; Schacht and Sommer, 2009). Personality plays an essential role in the assessment of individuals. Positive and negative personality assessments may correspond to the motivational systems for approach and avoidance, respectively. It has been suggested that negative personality signals a potential danger during social interaction, and the precedence given to this danger signal communicates the need to avoid harm (Williams et al., 2006). Given that, the amplified EPN for faces paired with negative personality in the present study may signal a selective, elaborate perceptual analysis of salient affective social information. It is worth noting that attention allocation has been argued to affect EPN amplitude (Adolphs, 2002; Schacht and Sommer, 2009). Schupp et al. (2007) proposed that more attention was allocated to emotional pictures, which enhanced EPN amplitudes. Accordingly, our observation of augmented EPN in the negative personality condition presumably indicated early implicit selective attention to distinctive faces that were related with negative personality information.

In short, both N170 and EPN showed stronger response to stimuli paired with negative compared to positive and neutral valence in the present study. There is a specious account for the personality effect on N170 and EPN that it reflects possible mediation from arousal of behavior describing sentences. Though stimuli arousal has been found to modulate early ERP amplitudes (Van Strien et al., 2009; Weinberg and Hajcak, 2010), with highly arousing stimuli elicit larger amplitudes than less arousing one (Schupp et al., 2003), this possible arousal interpretation is un-likely as the behavioral data suggest smaller arousal ratings for negative sentences than positive ones. Additionally, it is interesting to find comparable neural activity (N170 and EPN) for faces paired with neutral and positive personality. We speculate that this may be due to the concomitance of positive and negative information in the current study. It has been asserted that danger signals will engage the neural mechanisms for initial sensory input, whereas positive signals will be processed and will allow approach tendencies to proceed only once safety is assured (Williams, 2006).

### LPP and High-Level Elaboration of Affective Stimuli

In the present research, the LPP showed augmented amplitudes in response to faces paired with positive personality than those paired with neutral personality. These results contradict previous findings showing larger LPP to faces paired with negative information (i.e., Abdel Rahman, 2011). This inconsistency would ascribe to the stimuli differences. However, since the absence of detailed data about affective descriptions (e.g., arousal) in the study of Abdel, it becomes difficult to determine specific factors driven result differences. LPP, unlike N170 and EPN, is related to high-level, heightened cognitive elaboration of stimuli (for review, see Schupp et al., 2006). Thus it is often interpreted as a separate stage of encoding affective stimuli which can be affected by participants’ motivation or task demands (Langeslag et al., 2007; Rellecke et al., 2012). We suggest that the largest LPP response for faces associated with positive personality in the present study reflect sustained attention during the late period of facial processing was devoted in positive condition (Herbert et al., 2008). The faces paired with negative personality unexpectedly elicited smaller LPP amplitudes than did those with positive personality. The reduced amplitudes for faces in negative condition would reflect a shift of attention from negative to positive stimuli during the late stage of face processing. However, given the possible influence of stimulus arousal on the LPP component (Schupp et al., 2000), these findings could also be attributed to smaller arousal for negative descriptions than for positive descriptions. The elevated arousal for the positive stimuli is supposed to be due to the participants’ high involvement or emotional preference for virtue and positive personalities. Further investigation may counterbalance the stimulus arousal to address this issue.

The present study overcomes the limitations of previous studies to clarify the relationship between affective social information and visual processing, and further enrich research on social perception. Even though, some consideration should be given to its limitations and future prospects. First, it was not possible to evaluate gender difference due to the unbalanced sample in the present study, though some research suggests that this factor affects behavior (Levy et al., 2008). Therefore, future research should disentangle the gender influence. Second, the simplistic tasks used in our study resulted in high and comparable behavioral responses, which preclude us from obtaining explicit indexes of the personality effect. Future research should attempt a more sensitive task to further examine the influence of affective social information on facial processing.

## Conclusion

The present study has provided new electrophysiological evidence that affective personality information has an implicit influence on facial processing. Faces paired with varying personalities elicited distinct N170, suggesting an early automatic modulation of affective personality information on structural perception of facial stimuli. The personality influence continued to be evident in the subsequent EPN, indicating a further perceptual encoding of specific affective stimuli. The late stage of face processing indexed by LPP was also modulated by the context personality information, reflecting a high-level elaboration of emotional stimuli. More generally, these findings underline the ability of prior personality information, acquired from minimal information within a short period of time, to modify early and late neural representation of faces. N170 and EPN deflections showed similar patterns, with larger amplitudes for faces paired with negative personality than those with neutral and positive personalities. The facilitated processing of faces in the negative personality condition reflects an adaptive visual perception for negative stimuli. LPP showed a different pattern from N170 and EPN, with enhanced amplitudes for faces paired with positive personality, which was supposed to demonstrate sustained attention devoted in positive personality condition during the late period of face processing. Future research is warranted to determine whether the processing of specific facial features such as facial attractiveness is mediated by personality impression, which might further elucidate the mechanism of social impression.

## Author Contributions

QL: study design, data collection, data analyzing, paper writing; HW: data collection, data analyzing, paper writing; MD: data analyzing, paper writing; PH: data analyzing, paper writing; LM: study design; data analyzing; paper writing.

## Conflict of Interest Statement

The authors declare that the research was conducted in the absence of any commercial or financial relationships that could be construed as a potential conflict of interest.
