# Supplementary material for: Effect of Affective Personality Information on Face Processing: Evidence from ERPs
Source: Front Psychol. 2016 May 31;7:810. doi: 10.3389/fpsyg.2016.00810 (PMC4885863; doi:10.3389/fpsyg.2016.00810)
Supplement: Supplementary file 1 [file Data_Sheet_1.DOCX]

**Appendix**

*Behavioral descriptions used in the present research*

| Positive Description | Returned lost fortune to owner. |
| --- | --- |
|  | Bought food for homeless people. |
|  | Taught as a volunteer in West China. |
|  | Rushed into a fire to save people. |
|  | Went to the stricken area as a volunteer. |
|  | Saved a drowning child. |
|  | Escorted a disabled roommate to school. |
|  | Donated his/her bone marrow in hospital. |
|  |  |
| Neutral Description | Chatted with classmates. |
|  | Had dinner in a restaurant. |
|  | Shopped in a department store. |
|  | Took bike home. |
|  | Submitted resume on the net. |
|  | Gave a call to roommate. |
|  | Ate an apple. |
|  | Had a rest in the dormitory. |
|  |  |
| Negative Description | Played with the feelings of others. |
|  | Abused animals. |
|  | Betrayed friends for money. |
|  | Cheated on girlfriend/boyfriend. |
|  | Prescribed counterfeit drugs to patients. |
|  | Spoke ill of people everywhere. |
|  | Made mischief between people. |
|  | Treated his parents badly. |
